# Production of Bioethanol from *Artocarpus Integer* (Chempedak) Peel: An Approach Towards Clean Technology

**DOI:** 10.3390/nano16191259

**Published:** 2026-10-06

**Authors:** Yan Shan Ng, Asheild Clint Choisy, Kae Bin Lim, Zavier Ik Yang Jun, Benedict Raphael Soesanto, Sachin Sharma Ashok Kumar

**Affiliations:** 1 School of Engineering, Taylor’s University, 1 Jalan Taylor’s, Subang Jaya 47500, Selangor, Malaysia; ngyanshan03@gmail.com (Y.S.N.); asheildchoisy@gmail.com (A.C.C.); lkb2288@gmail.com (K.B.L.); zavier21k@gmail.com (Z.I.Y.J.); benedictraphaelsoesanto@gmail.com (B.R.S.); 2 Clean Technology Impact Lab, Taylor’s University, Subang Jaya 47500, Selangor, Malaysia

**Keywords:** bioethanol, biofuel, biowaste, clean technology, chempedak

## Abstract

This research focuses on a sustainable way to reduce waste and generate renewable energy by converting Chempedak bio-waste into bioethanol. It undergoes several phases: feedstock collection and pretreatment (mechanical, acid and alkaline pretreatment), hydrolysis, fermentation, and distillation. The main goal of this project is to yield ethanol from 300 g Chempedak peel waste by using a functioning and cost-effective way. When processing the project, the distillation process was then changed to ethanol evaporation which used a rotary evaporator to extract the ethanol, as it is more effective. The final product for the ethanol yielded 73% for 41 mL. This work provided solutions to society to reduce waste, promote more sustainable energy solutions, and increase economic growth in industries by finding more sustainable options.

## 1. Introduction

Biowaste is the biodegradable organic waste produced by humans; this can be from the animals and plants we consume or also the products we use that may produce waste products. Solid Waste Management and Public Cleansing Corporation have conducted an analysis showing that there are about 39,078 tons of waste produced daily in Malaysia, which amounts to 1.17 kg per person [1]. According to some reports, more than 30% of waste is disposed of as food waste, while other reports state that organic solid waste makes up 57% of municipal solid waste [2]. Currently, most of the waste in Malaysia ends up in landfills, and the others end up incinerated, with only 17.5% of it recycled [3]. Despite being biodegradable, these wastes may affect the environment and degrade soil quality if they are not properly treated. They can also produce methane, which may harm the environment if it is not stored. The waste in landfills will release toxins from the biowaste, and these toxins will lead to the soil or water which will pollute them [4]. Hence, the biowaste could be transformed into valuable products like bioethanol instead of being discarded.

Bioethanol can be produced from these biowaste materials since they include monomeric sugars that are either in monomeric form or are still in polymer form. These polymers can be processed to form glucose, which is the main component in making bioethanol. The cost of producing bioethanol is decreased by this method’s relative simplicity. It can move away from traditional fossil fuels that pollute the atmosphere by using bioethanol as fuel.

The gasoline used in cars is made with blended fuels which contain 90% gasoline and 10% ethanol [5]. Depending on the kind of vehicle, this composition can differ. For instance, E85, which contains up to 85% ethanol, can be used in flexible fuel vehicles [6]. The flexibility provides consumers with options to use higher renewable fuel content, contributing to reduced fossil fuel dependency. Bioethanol that is produced by biowaste can be used in this way, which in turn up-cycles the waste. The conversion of biowaste into bioethanol helps to address the global waste problem by diverting organic materials from landfills and reducing methane emissions from anaerobic decomposition while simultaneously creating a renewable energy source. Moreover, bioethanol serves as an excellent alternative to traditional fossil fuels, as it burns cleaner than other hydrocarbons, significantly reducing harmful air pollutants. When combusted, ethanol emits fewer nitrogen oxides, hydrocarbons, and carbon monoxide compared to gasoline, leading to improved air quality and reduced smog formation. Cars that use ethanol emit no net carbon emissions due to ethanol’s lower combustion temperature [7]. Additionally, some studies indicate that the second-generation bioethanol derived from biomass can reduce carbon emissions by 90% [7]. To determine how an automobile can run entirely on ethanol, programs such as ETHANOL 100 have also been launched [8]. The need for bioethanol is growing as ethanol-fueled technologies are developed worldwide, which opens up the possibility of turning biowaste into bioethanol.

There are several challenges in biowaste conversion, which include variability of biowaste, lack of efficiency throughout the process, high initial cost, and lack of policy and incentives support. First, the variability of biowaste is one of the biggest challenges, as biowaste consists of food, agriculture residue, and kitchen waste or other waste. Each of these components contains distinct chemicals, some of which may not be suitable for use in bioethanol production. Furthermore, the pretreatment processes needed for these various chemicals varies, which could affect the components of the others. As a result, the process’s efficiency is decreased.

Next, the overall lack of efficiency throughout the process mostly stems from the pretreatment, which can be highly energy intensive. This is particularly true for mechanical pretreatment, as it might require a lot of energy to break down biowaste into tiny pieces. Other pretreatments, including chemical pretreatment, are less expensive, but they also have neutralization issues and can be harmful if improperly handled.

Moreover, the high initial cost of the specialized equipment is needed, as there are many different components needed in bioethanol plants. This may result high costs, especially for initial capital, which makes it difficult for a plant to be built. Additionally, the incentives for doing so are low, as landfills are often cheaper than bioethanol production. There are also no policies which can encourage businesses to adopt biowaste to bioethanol conversion. Hence, the lack of incentives and policy support makes converting biowaste into bioethanol less attractive compared to cheaper methods like landfill disposal.

The recent literature continues to demonstrate the broad feasibility of bioethanol from fruit-waste feedstocks. Ref. [9] reviewed bioethanol production from pineapple, mango, pawpaw, and watermelon wastes, showing how the substantial peel and pulp volumes generated during fruit processing can be converted into ethanol via enzymatic hydrolysis, fermentation, and distillation while noting that seasonal availability and substrate variability remain key challenges to process optimization. Ref. [10] complied results across tropical fruits, citrus fruits, and berries, reporting bioethanol yields of up to 26.84 g/L from banana peel alone and up to 22.77g/L when orange peel is combined with rice husk waste, illustrating both the scale of the potential recovery and the value of co-substrate strategies. Most recently, ref. [11] compared mango, orange, and banana residues using green ionic liquid pretreatment and identified mango residue as the most promising feedstock, owing to its high sugar content and favorable cellulosic composition. Fruit peel waste is therefore an increasingly active area of bioethanol research; however, feedstocks endemic to southeast Asia such as Chempedak remain comparatively underexplored, underscoring the contribution of the present study in extending this body of work to a locally abundant tropical fruit waste stream.

One tropical fruit that is commonly found in Southeast Asia in Malaysia, Thailand, Myanmar, Indonesia, and Vietnam is the Chempedak (*Artocarpus integer*). The significant waste of the Chempedak is its peels, which have a thin greenish-yellow-color skin dotted with pentagons [1]. However, Chempedak contains 70–80% non-edible parts with a weight of 5–7 kg per fruit, which includes the peel and other central parts. These wastes are high in carbohydrates, up to 76% [12]. Thus, one advantage of Chempedak is the existing soluble sugar of 19.75%, which can be obtained without much processing, and an extra 27.75% cellulose and 4.12% starch, which need more processing [13]. Chempedak peel has carbohydrates within it and can be fermented by yeast to produce ethanol and carbon dioxide [13].

This study provides a solution to both waste management and the production of alternative energy while attempting to solve the problems and challenges that may happen when converting biowaste to biofuel. Additionally, this project provides a secondary goal of optimizing the process and finding optimal methods to increase efficiency in the future.

## 2. Materials and Methods

### 2.1. Materials

The materials and chemicals used in this experiment can be seen in Table 1.

### 2.2. Pretreatment

To produce ethanol, Chempedak or *Artocarpus integer* peel is used. This is due to its abundance in Malaysia as well as its peel being considered waste after eating. Although this peel is considered waste, a lot of sugars, cellulose, and starch is present in the Chempedak: approximately 19.75 ± 0.16%, 27.75 ± 0.06%, and 4.12 ± 0.02% [13]. These sugars, however, are not accessible for ethanol fermentation yet, as they are not in their monomeric glucose form.

The sugars found in cellulose are bonded by beta-1–4 glycosidic bonds and are used for the fruit cell walls [14]. Thus, they give the fruits a strong and rigid shape along with lignin and hemicellulose, which are also found in cell walls. To be able to access these sugars, pretreatment will be performed, as it breaks down the cell walls, which allows the sugars found within it to be accessible as well as the cellulose itself to turn into smaller sugar polymer chains.

#### 2.2.1. Mechanical Pretreatment

Mechanical pretreatment was performed by drying the Chempedak peel to remove its moisture content and grinding it to smaller pieces. Firstly, the Chempedak was cut to smaller pieces to be able to be placed in the oven. Initially, the peel was put in an oven for 60 °C for 6 h, as stated in a study [15]. However, the peel was still moist; thus, the peel was put in the oven for 120 °C for 2 h, which allows moisture to evaporate while the sugars are not burnt, as they decompose at 180–185 °C [16]. The peel was then placed in a mechanical grinder and was sieved to obtain a size of 1 mm [17]. A total of 300 g of this powder was collected to undergo three different pretreatment processes.

#### 2.2.2. Acid Pretreatment

Acid pretreatment is then performed to break down the previously mentioned hemicellulose bonds, allowing sugar to be processed further, the hemicellulose bonds being sugars as well [18,19]. The powder is split into two, with 100 g each, and is mixed into a sulfuric acid (H_2_SO_4_) solution of (2% *w*/*w*) and is placed for 2 and 3 h at 160 °C each [20,21]. This amounts to 200 mL of 1 M sulfuric acid solution.

After being treated, 400 mL of sodium hydroxide (1 M) was added to neutralize the solution to the pH of 4.8 as the next process, hydrolysis, requires a pH of 4.8. Since the hemicellulose is broken, some sugars will be diluted in the water; thus, neutralization was performed instead of washing to not lose the sugar found within it [18]. The pH was then tested with a litmus paper to ensure that it is within range.

#### 2.2.3. Alkaline Pretreatment

Unlike acid pretreatment, alkaline pretreatment primarily targets the removal of lignin, as it dissolves within the alkaline solution [22]. By doing so, the sugars will be more exposed when put through hydrolysis. Firstly, the sodium hydroxide solution is prepared by mixing the 4 g of sodium hydroxide into a 100 mL solution to obtain a 1 M solution with its sodium hydroxide content being 4% (*w*/*w*). This is then heated for 1 h at 120 °C [23,24].

Since the sugars and hemicellulose are not broken down, they remain within the solid phase of the solution, thus washing is performed until the liquid within the solution is clear. The pH is also measured until it reaches the pH of 7 [13].

### 2.3. Hydrolysis

Hydrolysis refers to a process where the sugars present will be broken down to simpler forms, mainly monomeric glucose, which can be fermented. By doing pretreatment, the hydrolysis process happens faster, as these sugars are more accessible for the enzymes and have a higher yield of sugar, as the other sugars that hydrolysis cannot break down are broken down by the pretreatment process. This is conducted with the help of an enzyme known as cellulase, which breaks down the cellulose in the Chempedak peel. This enzyme is a combination of many enzymes which all aid in breaking down the different sugars found inside the Chempedak peel; the main enzymes are exoglucanase, glucosidase, and endoglucanase [25].

The initial bonds that are broken down by the exoglucanase and the endoglucanase are those within and between the fibers of the Chempedak peel, which are formed by interactions between OH molecules. The exoglucanase breaks down the reducing bonds, mainly aldehyde or ketone bonds, as well as the non-reducing bonds, which form a crystalline structure mainly consisting of acetals. This process, however, is mostly accomplished when pretreated [25].

The second process, on the other hand, involves the glucosidase, which converts the shorter cellulose polymers into monomeric glucose [25].

### 2.4. Buffer and Enzyme Preparation

A buffer solution was prepared for the alkaline pretreatment, as its pH is 7, which is not optimal for cellulase, which has an optimum pH of 4.8. The buffer solution prepared was a citric buffer solution consisting of 5 g of citric acid and 7.5 g of sodium citrate dihydrate with a molarity of 0.05 M. Due to storage constraints, 1 L of the solution was made, unlike the proposed solution of 1.5 L, where the buffer will be placed in 800 mL and the alkaline pretreated solid phase is diluted to 200 mL.The prior citrate buffers were prepared following the calculations in ref. [26].

A cellulase solution is prepared by adding 100 mL to the amount of cellulase needed. Initially, impurities such as starch were expected to be present, thus it was refrigerated to separate the starch. However, there was no starch present in the cellulase we used, allowing the cellulase solution to be added to the pretreated solution. Since the units of the enzyme are unknown, it was assumed that there are 10,000 U/g in the cellulase used.

During hydrolysis, an excess of enzymes will only quicken the process and will have no drawbacks. Since the units of the enzyme are unknown and there is no disadvantage in adding excess cellulase, a total of 10 g of enzyme was placed in the solution.

The 3 solutions are then put in an incubator at 50 °C for 72 h. This was chosen as it is the optimum temperature for the enzyme and is the optimum time to fully hydrolyze the solution [24]. Two samples of each solution were taken every day to find the ideal time for the solution to hydrolyze.

### 2.5. Fermentation

Once glucose is obtained, the solution undergoes fermentation to produce ethanol. This process uses *Saccharomyces cerevisiae*, a fungus used when brewing alcohol. It grows best in acidic conditions with a pH between 4 and 6 and at temperatures ranging from 30 °C to 35 °C [27]. Equation (1) shows the reaction as below,

(1) $${C_{6}H_{12}O}_{6}\left(aq\right)\underset{yeast}{\to }{2C}_{2}H_{5}OH\left(aq\right)+{2CO}_{2}$$

For each solution, 1 g of yeast is added, and the container is sealed with a seal connected with a pipe to a beaker filled with water to see the carbon dioxide being produced. The seal is used to ensure anaerobic conditions, which are crucial for ethanol production. If oxygen is present, microbes will break down carbohydrates more efficiently for energy, reducing ethanol production, as they prioritize aerobic respiration [28].

The solution was placed in an incubator at a pH of 4.8 with a temperature of of 30 °C for 7 days [17,29]. Like hydrolysis, excess yeast will help speed up the reaction and will have no negative effect on the process. Thus, 10 g of yeast is added to the solutions.

### 2.6. Extraction

After fermentation, most of the glucose monomers have been converted into bioethanol. However, the solution remains impure, containing spent yeast, salt, water, and other organic compounds from the peel. Thus, distillation is required to extract the ethanol found. Traditionally, a distiller is used to obtain ethanol; it works by utilizing the difference in boiling points between ethanol and water. Ethanol, with a lower boiling point of 78.37 °C, evaporates first when the solution is heated to around 80 °C, below water’s boiling point of 100 °C. Due to limitations within our lab, the distiller was not able to be used; thus, a rotary evaporator is used instead, which works under a similar premise. In this scenario, the rotary evaporator provides an advantage, as its temperature is more controlled as well as its rotation of the flask, allowing a more even heat distribution and leading to a more precise extraction process. Due to this, less cycles of extraction were needed during the experiment.

A rotary evaporator also uses the difference in boiling points to extract ethanol, but it is conducted under a vacuum, lowering the boiling point of the solution. In this case, the temperature of the water bath was at 60 °C with a vacuum of 175 mbar, allowing the ethanol vapor to be at 40 °C, with the condenser being set at 20 °C, allowing the ethanol vapor to travel to the receiving flask [30].

### 2.7. Data Collection and Analysis

The final ethanol concentration was tested with a refractometer and hydrometer. The refractometer uses how much the sample refracts to measure its refractive index to find its concentration [31]. Next, a hydrometer is used, where the solution can be placed in a graduated cylinder. The hydrometer used measures the alcohol percentage directly and works by the different specific gravities that are found with different alcohol percentages [32]. Additionally, testing was performed on the glucose levels after hydrolysis with Benedict’s Solution to find which pretreatment method yielded the most glucose.

### 2.8. Glucose Testing

After hydrolysis, the resulting solution should contain sugars in monomeric form or glucose. This can be tested using Benedict’s reagent and will be performed on three solutions to determine which yields the most glucose. Benedict’s reagent is prepared by mixing 50 mL of an 8.7 g copper (II) sulphate pentahydrate (CuSO_4_·H_2_O) solution with 400 mL of a solution containing 86.5 g sodium citrate and 50 g sodium carbonate, which is then diluted to 500 mL [33].

To determine glucose concentration, 3 mL of Benedict’s solution is mixed with 3 mL of the sample solution in a test tube. The mixture is heated in boiling water for 5 min and allowed to cool until the precipitate settles. The glucose concentration is indicated from the solution’s color, with red indicating the highest glucose content and blue showing no glucose present [34].

### 2.9. Refractometer Test

The refractive index of the distillate from the extraction was measured using the DR-A1, ATAGO Abbe Refractometer to determine its ethanol concentration. The Abbe refractometer uses the principle of prism refraction to measure the refractive index of solutions [35]. By observing and aligning the refraction boundary line with the crosshairs, the refractometer displays the measurement value of the sample’s refractive index and Brix percentage along with its temperature on the digital display screen [36]. To enhance accuracy, a calibration graph was created using standard ethanol solutions before measuring our samples. This approach ensures precise and tailored measurements while minimizing the reliance on generic data.

Before testing the distillate, a calibration graph of refractive index versus ethanol concentration was measured and plotted using refractive index with known ethanol concentrations of 20%, 40%, 60%, 80%, and 95% respectively, as depicted in Figure 1. This calibration graph serves as the guideline for determining the ethanol concentration in each of the distillates.

The refractometer has a few limitations, one of which is that the refractive index has a similar value from the 65 and 95% mark, as it forms a parabola, leading to potential inaccuracies in that range, as it has two ethanol values for the same refractive index within the 65 and 95% range. Thus, a hydrometer test was conducted to determine the value more accurately alongside the refractive index to approximate the ethanol value better.

### 2.10. Alcohol Hydrometer Test

The ethanol concentration of the distillate from the extraction was measured using an alcohol hydrometer. The alcohol hydrometer uses Archimede’s principle of buoyancy to measure the specific gravity of alcoholic solutions, which is directly proportional to its concentration [37]. By immersing the alcohol hydrometer into the sample, the concentration of ethanol can be directly determined by reading the value indicated on the hydrometer at the point where it aligns with the surface of the sample solution. This is because alcohol hydrometers are already pre-calibrated to directly display ethanol concentration based on the solution’s specific gravity.

However, due to the potential of other compounds within the solution, the hydrometer’s result may not be 100% accurate, as other compounds may affect the density, thus both tests will be used. The scientific theory of the test method will be elaborated in the following section.

The alcohol hydrometer used in the lab consists of a set of 3 hydrometers, where the 1st alcohol hydrometer has an alcohol concentration scale from 0% to 40%, the 2nd hydrometer has scale from 40% to 70%, and the 3rd hydrometer has a scale from 70% to 100%. Depending on the concentration, all three were used in this experiment to find which scale represents the alcohol concentration.

### 2.11. Overall Procedure

The overall process conducted will follow the flow chart below, as shown in Figure 2.

## 3. Results and Discussion

### 3.1. Theoretical Yield

The theoretical final yield of ethanol depends on how effective each step is from pretreatment to extraction. The theoretical yield will assume complete breakdown of lignin and cellulose from pretreatment, complete hydrolysis of the sugars into glucose, complete fermentation for every molecule of sugar, and no losses during distillation. This also assumes the composition of Chempedak as shown in Table 2 [13]:

When converting the composition to glucose and then to ethanol, its molecular weight will be used to find the final calculated weight and volume of ethanol. The calculated result can be seen in Table 3.

### 3.2. Glucose

A glucose test was conducted using Benedict’s solution, where its color would indicate the concentration of glucose within the solution. Visually, all the acid pretreatments with different time intervals resulted in a brick red color, which indicates the concentration of 1 M of glucose, as illustrated in Figure 3. As for the alkaline pretreatment, the time intervals also did not affect the color, resulting in a concentration of around 0.2 M of glucose, as illustrated in Figure 4.

Since the acid pretreatment between 2 and 3 h and the different time intervals produce similar results, which are around 1 M in glucose concentration, all the calculations for the samples will be performed the same.

As seen in the results, the test being performed is not representative, as 270.234 g of glucose is not possible within a 100 g sample of biomass. In addition to that, the results may be inaccurate, as the solution has a natural brown-red color, which may affect the color, thus making the brick red color originate from the biomass rather than the reaction from the test. Therefore, the glucose test is unsuccessful and inconclusive; however, it may be concluded that the acid pretreatment broke down the most amount of sugar due to a darker brick red solution forming.

This is due to the content within the Chempedak peel, which consists of up to 23% pectin and 13% hemicellulose compared to 23% lignin. As mentioned before, acid pretreatment breaks down hemicellulose as well as pectin, whereas alkali pretreatment targets lignin; thus, acid pretreatment can break down more components into sugar compared to alkaline pretreatment [18,19,22].

### 3.3. Extraction Sets

Multiple extractions were conducted to obtain a higher yield and higher amount of ethanol. The sets of extractions are stated below.

Extraction Set 1

- 1st Distillate: distillate from 2 h of extraction of fermented solution.
- 2nd Distillate: distillate from 2 h of extraction of distillate 1.
- 3rd Distillate: distillate from 1 h of extraction of distillate 2.
- 4th Distillate: distillate from 0.5 h of extraction of distillate 3.

Extraction Set 2

- 1st Distillate: distillate from 3 h of extraction of 1st set extraction distillate 1 waste.
- 2nd Distillate: distillate from 2 h of extraction of distillate 1.

Extraction Set 3

- Combination of 4th distillate from extraction set 1 and 2nd distillate from extraction set 2
- 1st Distillate: distillate from 1 h extraction of set 3
- 2nd Distillate: distillate from 0.5 h of extraction of distillate 1.

### 3.4. Refractive Index Test

The refractive index values of the distillates obtained were calculated using Equation (2) below to determine ethanol concentration. The results are tabulated below.

(2) $$y=\left(-3\times 10^{-8}\right)x^{3}+\left(9\times 10^{-7}\right)x^{2}+0.0005x+1.3331$$

As shown in Figure 5, some results are doubled, such as the 4th distillate of extraction set 1 and the 2nd distillate of extraction set 2; this is due to the quadratic equation which dictates its concentration, resulting in multiple results for certain datasets. Due to the azeotropic nature of water and alcohol mixture, it can be assumed that the lower percentage or the one presented was acquired.

### 3.5. Alcohol Hydrometer Test

The ethanol concentration of distillate was directly read from the scale on the alcohol hydrometer where it aligns with the sample’s surface. Additionally, both extractions’, set 1 and set 2, distilled waste was measured to ensure that there was no ethanol content in it.

Unfortunately, the alcohol hydrometer was obtained only after completing extraction set 1 and set 2. Therefore, we were only able to measure the ethanol concentration of the final distillates from extraction set 1 and set 2. This limitation was also due to the small quantity of distillate obtained in each round from set 1 and set 2. They were directly used in subsequent extractions to avoid wastage, leaving no individual distillates for separate measurements. However, for extraction set 3, both distillates were successfully measured with the alcohol hydrometer, providing the final few results for this extraction process. The measurement of results is depicted in Figure 6 and Figure 7 respectively.

### 3.6. Comparison Between Refractive Index Test and Alcohol Hydrometer Test

Although the refractive index test provides a more comprehensive dataset, it has significant limitations that impact its result’s accuracy. This test involves manual alignment of the shadow boundary with the crosshairs using the adjustment knob. Additionally, the differences in refractive index values for ethanol concentrations can vary by as little as 0.0001, making the test highly sensitive to small errors. Despite these limitations, the refractive index test produced a final concentration result of 72.95%, which closely aligns with the alcohol hydrometer test result of 73.0%, validating the reliability of the hydrometer readings.

In contrast, the alcohol hydrometer test eliminates the need for manual adjustments and complex polynomial calculations. Although it is calibrated for ethanol, its mechanism does not allow for extremely precise results, as it assumes of a mixture of mostly pure ethanol and water. However, there are other compounds within the mixtures, such as volatiles, which may affect the overall specific gravity of the liquid, resulting in slightly off values.

In conclusion, the alcohol concentration selected was 41 mL of 73% ethanol (29.93 mL of ethanol), as this result was obtained by both the hydrometer and refractometer. This indicates that 29.93 mL ethanol is present in the final solution, which validates the success of the whole Chempedak-to-bioethanol production process.

### 3.7. Analysis

After finding the total amount of ethanol obtained, to test the effectiveness of the process, its theoretical maximum yield will be compared to its actual yield, and its yield efficiency will be obtained by dividing the actual ethanol obtained by the maximum theoretical volume. As shown in Table 4, the yield efficiency was found to be 29.818%, indicating that the methods chosen were able to produce 29.818% of the theoretical maximum. This relatively low value may be due to various reasons such as inaccuracies in calculated ethanol volume, as the exact amount of glucose within the Chempedak peel was not tested but was instead estimated. Another reason could be due to the mixing of both pretreated solutions together, as the alkali-pretreated mixture had a lower glucose concentration compared to the acid-pretreated mixture. Due to the nature of the project, this was required; however, to better observe the effectiveness of the pretreatment process, both should be isolated and tested separately. Nevertheless, this experiment has achieved its goal of the conversion of bioethanol from Chempedak waste.

## 4. Failure Analysis and Limitations

Systematic error is a consistent difference between the obtained value and the actual value of something. It shifts data from their actual values, resulting in false conclusions. During the feedstock collection and pretreatment stage, contaminants such as dirt and unclean water may be present. To mitigate this issue, proper cleaning techniques, including controlling the number of washes and using a consistent and appropriate amount of water, were implemented.

In the mechanical pretreatment step, feedstock loss may occur during the transfer from the grinder to the beaker. This loss can reduce overall yield and cause inaccuracies in calculations. To minimize this issue, the transfer pathway from the grinder to the beaker should be enclosed to prevent spillage.

For the chemical pretreatment process, uneven mixing can lead to suboptimal chemical reactions and incomplete treatment. This can be improved by using an overhead mechanical stirrer to ensure uniform mixing throughout the reaction. Additionally, residual alkaline may not be fully washed or neutralized out after pretreatment. This can be mitigated by washing the feedstock and confirming neutrality through pH measurements.

During the hydrolysis process as well as the buffer and enzyme preparation, enzymes may be used under non-optimal temperature or pH conditions, which reduces effectiveness. To prevent this, the mixture should be constantly monitored to ensure optimal pH and temperatures.

Similarly, on the fermentation process, non-optimal temperature, pH, or oxygen levels will reduce the amount of fermentation occurring, as the yeast will use the oxygen present instead. The use of an incubator can help maintain optimal temperature, and ensuring proper sealing will ensure anaerobic conditions.

Additionally, experiment procedures can be improved to optimize in finding the true amount of ethanol produced for each pretreatment, as this experiment fails to do so due to the project scope requirements of maximizing yield and not a comparison. Along with that, testing can be improved for glucose, as the result obtained was not possible; this can be conducted by dilution to ensure other precipitates do not take over the brick red color. Different tests are able to quantify the glucose levels, such as glucose test strips or specific refractometers.

Lastly, random errors may also occur on testing and procedural equipment, such as an inaccurate temperature and pH reading. This can be mitigated by repeating the experiment multiple times to ensure consistent results.

## 5. Future Perspective

The success of our small-scale bioethanol production serves as a foundation for future advancement in turning biowaste into renewable energy. From our experiment, we have found various alternatives to convert Chempedak peel to bioethanol, with some producing better yield than others. Refining and optimization will be essential in improving yields and lowering production cost. If there is more capital, more experiments can be conducted to find the best steps to each process, such as enhanced pretreatment methods, increased enzymic efficiency, and more.

Thus, bioethanol’s versatility allows it to be used across many industries, including in cosmetics, healthcare, food and beverage, automotive, and pharmaceutical sectors, expanding its market potential. In the future, this system could be integrated with other renewable energy sources to create a diversified and sustainable energy source. With continued research and development, production of bioethanol from biowaste can contribute significantly towards building a more sustainable future and circular economy.

## 6. Conclusions

In conclusion, this project addresses the challenges of biowaste management by focusing on converting Chempedak (*Artocarpus Integer*) peel into bioethanol, which is a renewable energy source. By employing a structured design-thinking approach, we refined each step of the ethanol production process, including feedstock collection and pretreatment (mechanical, acid, and alkaline pretreatment), hydrolysis, fermentation, and ethanol extraction.

The key findings of this project include the successful optimization of mechanical pretreatment by turning Chempedak peel into smaller pieces and then into powder form of size 1 mm^3^ and dehydrating in an oven at 120 °C for 2 h, which effectively removed moisture while preserving sugars. Acid pretreatment proved more effective than alkaline pretreatment in enhancing sugar availability for hydrolysis. Fermentation using yeast in sealed containers efficiently produced ethanol, as indicated by active carbon dioxide generation. Additionally, ethanol recovery was significantly improved using a rotary evaporator operating at 60 °C under vacuum, resulting in a final ethanol concentration of 73% and a recovered volume of 41 mL, equivalent to 29.93 mL of 100% ethanol, a yield efficiency of 29.83% against the theoretical maximum. This efficiency also reflects the process’s current limitations: the theoretical maximum was calculated from the literature composition values rather than a directly measured glucose content since Benedict’s reagent, the only glucose test available in this study, proved unreliable for quantification and could only compare pretreatment quantitatively. The acid- and alkaline-pretreated fractions were also combined before final extraction, which limited the ability to isolate each pretreatment’s individual contribution to yield. Our method is unique because it turns an environmental burden into a useful resource by using Chempedak peel. When compared to traditional approaches, our process integrates cost-effective mechanical advances and scalable extraction processes, making it for greater commercial applicability. 

This research aligns with multiple United Nations Sustainable Development Goals (SDGs). It contributes to SDG 7 (Affordable and Clean Energy) by generating bioethanol to reduce fossil fuel dependency, supports SDG 12 (Responsible Consumption and Production) by transforming waste into valuable energy, and advances SDG 13 (Climate Action) by mitigating methane emissions and providing cleaner combustion fuels.

Through the management of biowaste and the creation of renewable energy, this project promotes environmental responsibility and lessens reliance on landfills while providing sustainable energy options that residents and businesses may use. Along with lowering carbon footprints and fostering economic opportunities, it improves societal well-being. This project demonstrates innovation in sustainable technology by integrating biowaste management with renewable energy production. It reinforces the potential for bioethanol derived from Chempedak peel waste to support the circular economy, inspiring future research and commercialization in clean energy solutions.

## Figures and Tables

**Figure 1:**
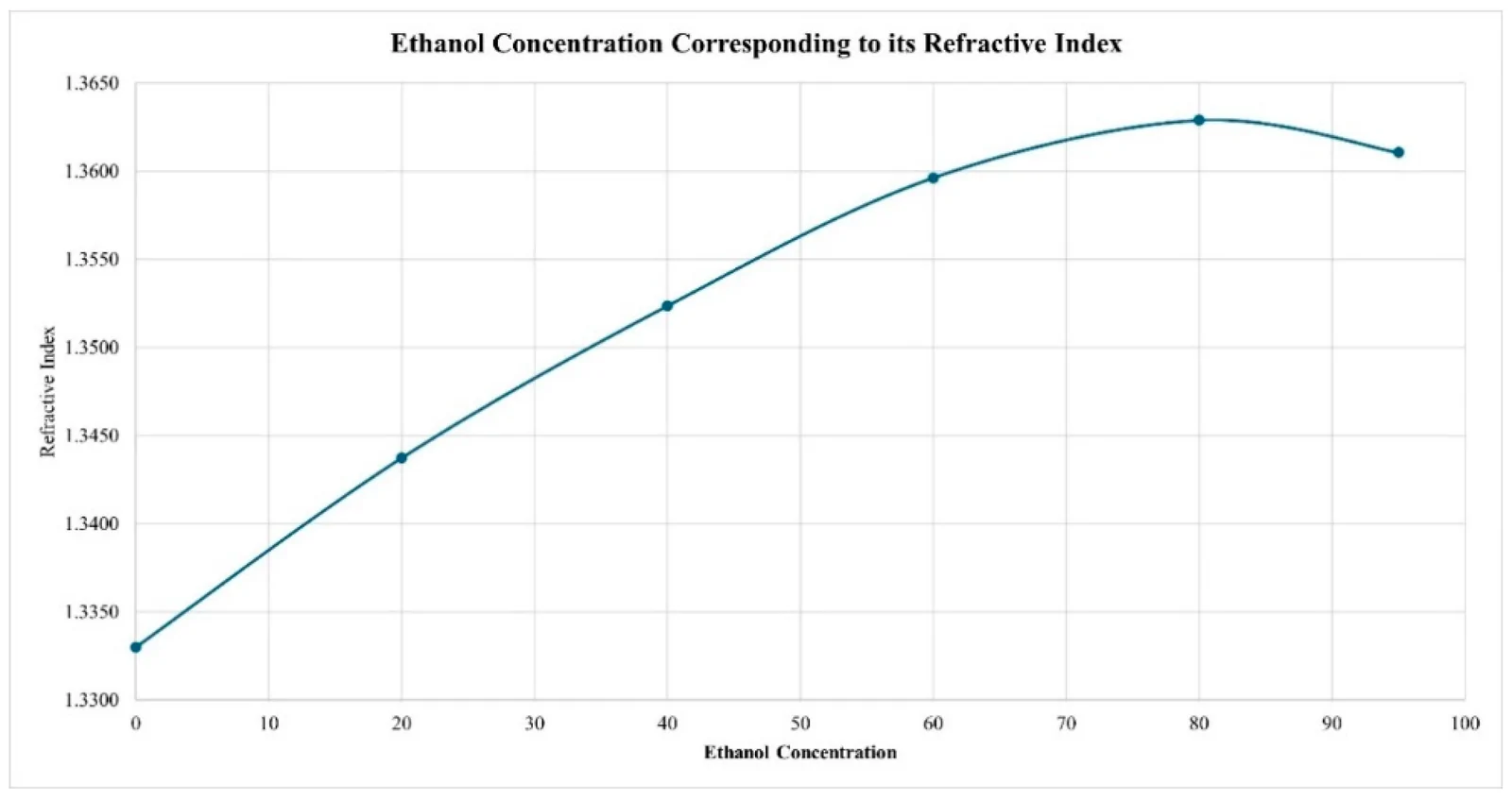
Graph of ethanol concentration corresponding to its refractive index.

**Figure 2:**
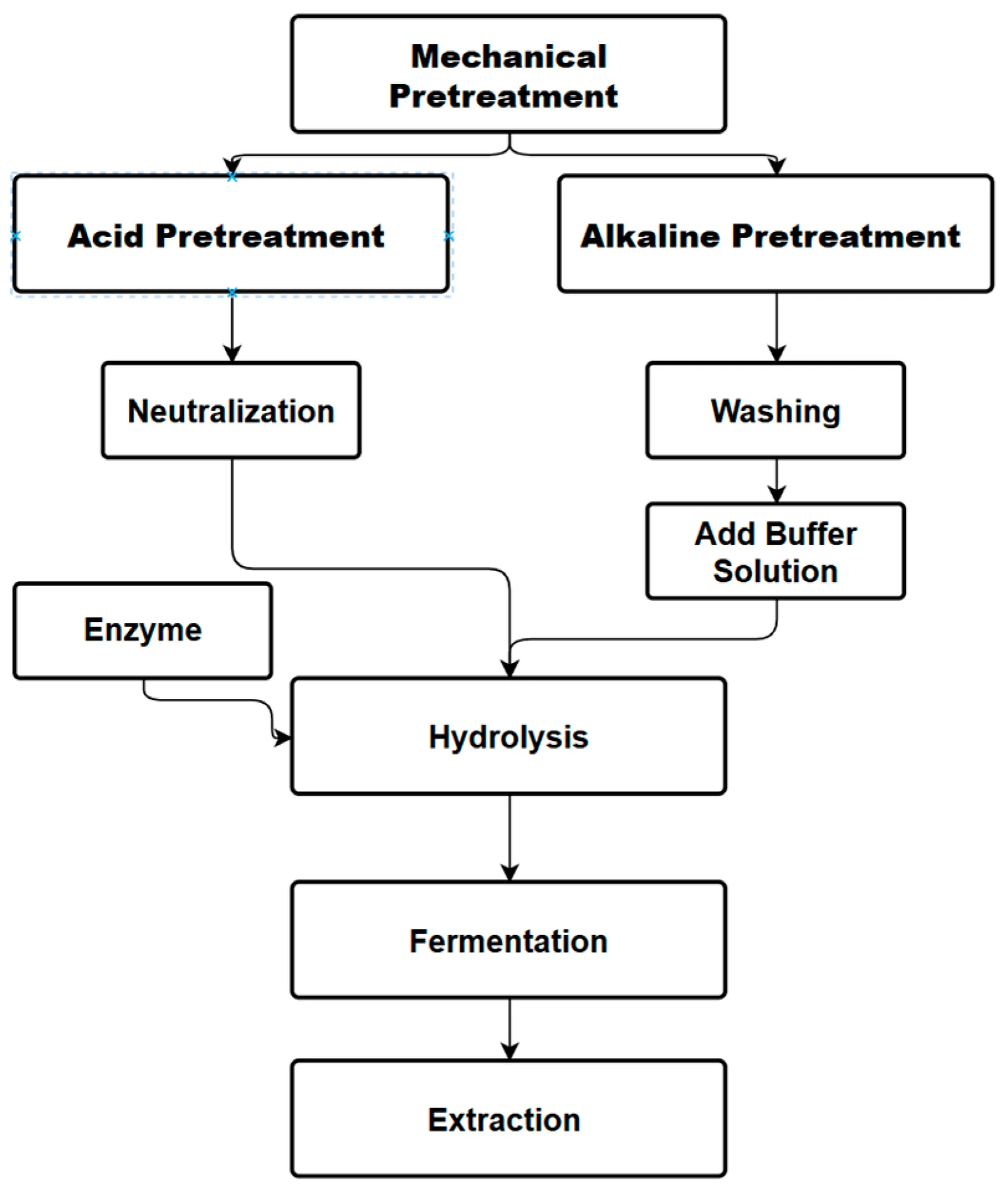
The procedure followed by the authors to produce bioethanol.

**Figure 3:**
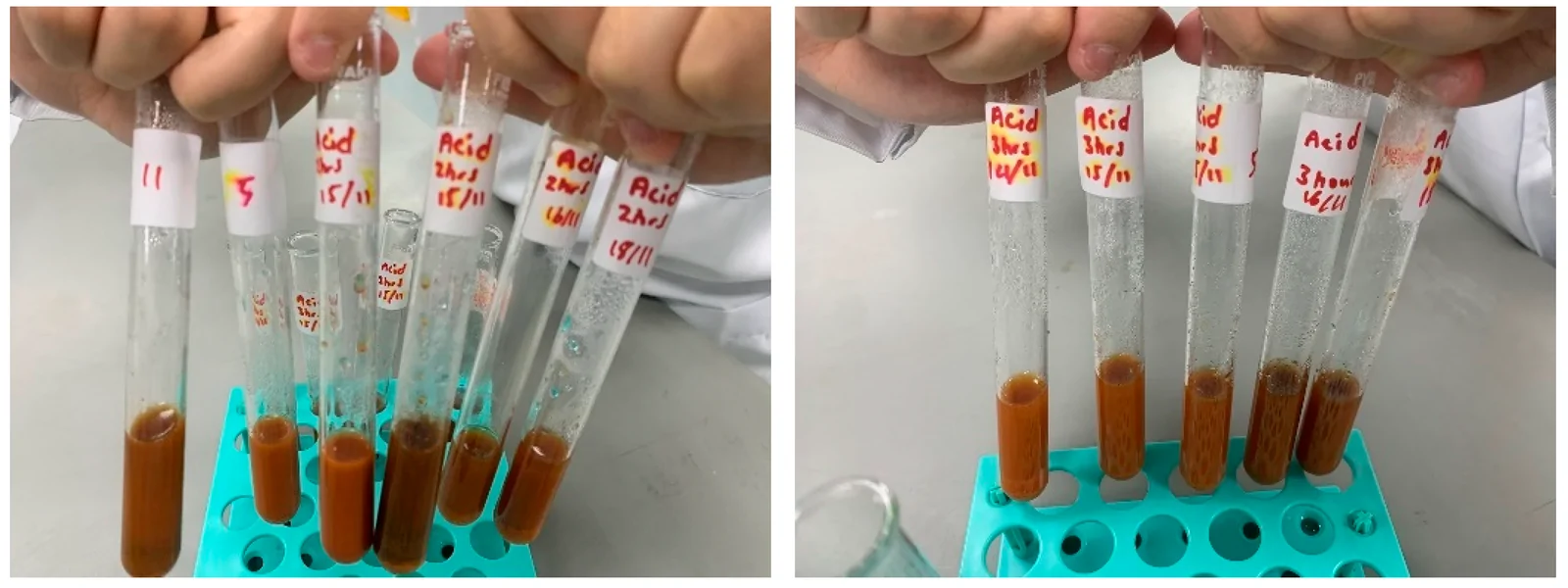
Benedict’s test on acid-pretreated solution indicating high glucose content due to the opaque brick red color forming.

**Figure 4:**
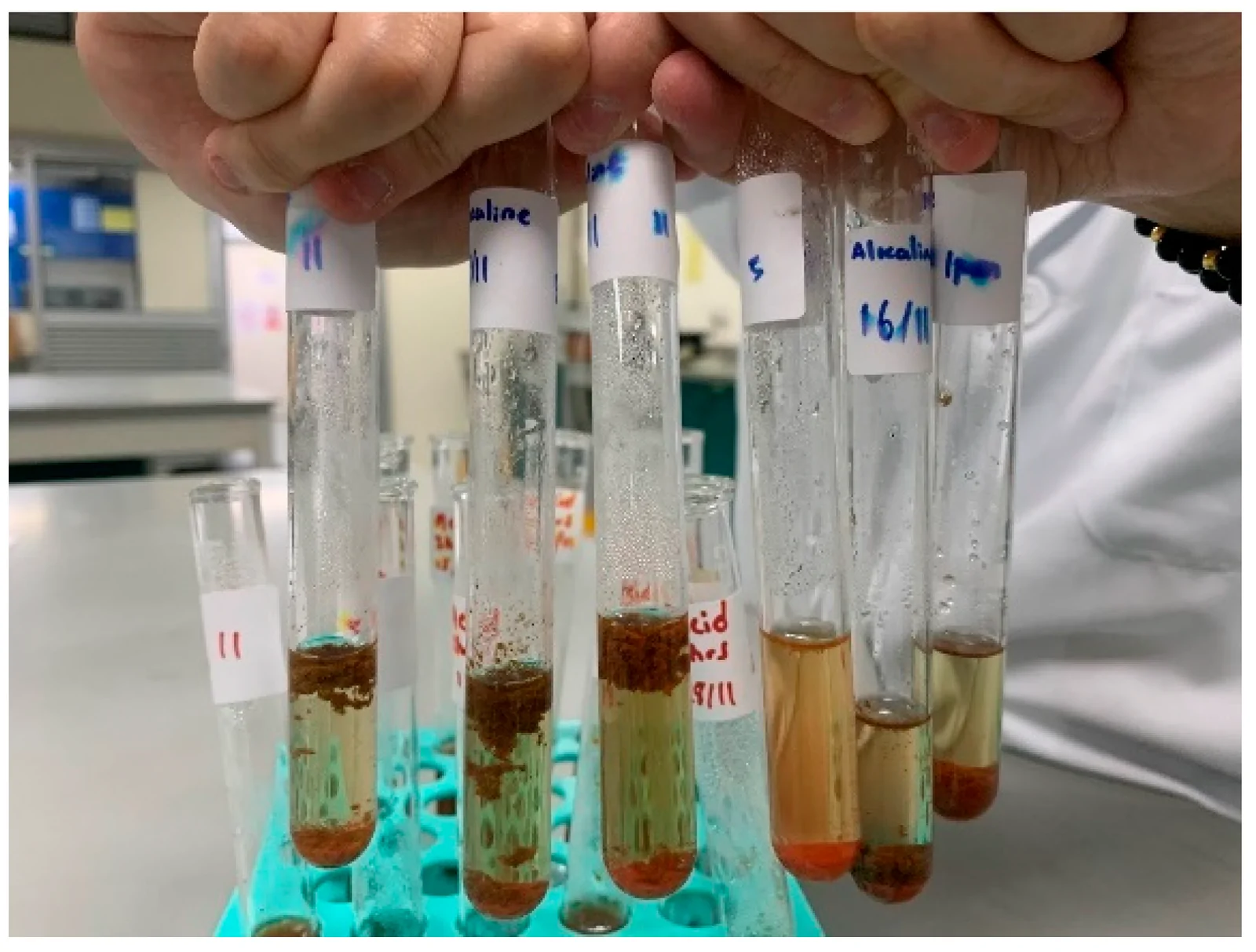
Benedict’s test on alkaline-pretreated solution indicating high glucose content with its translucent red brick color; however, separation of the solution was also observed.

**Figure 5:**
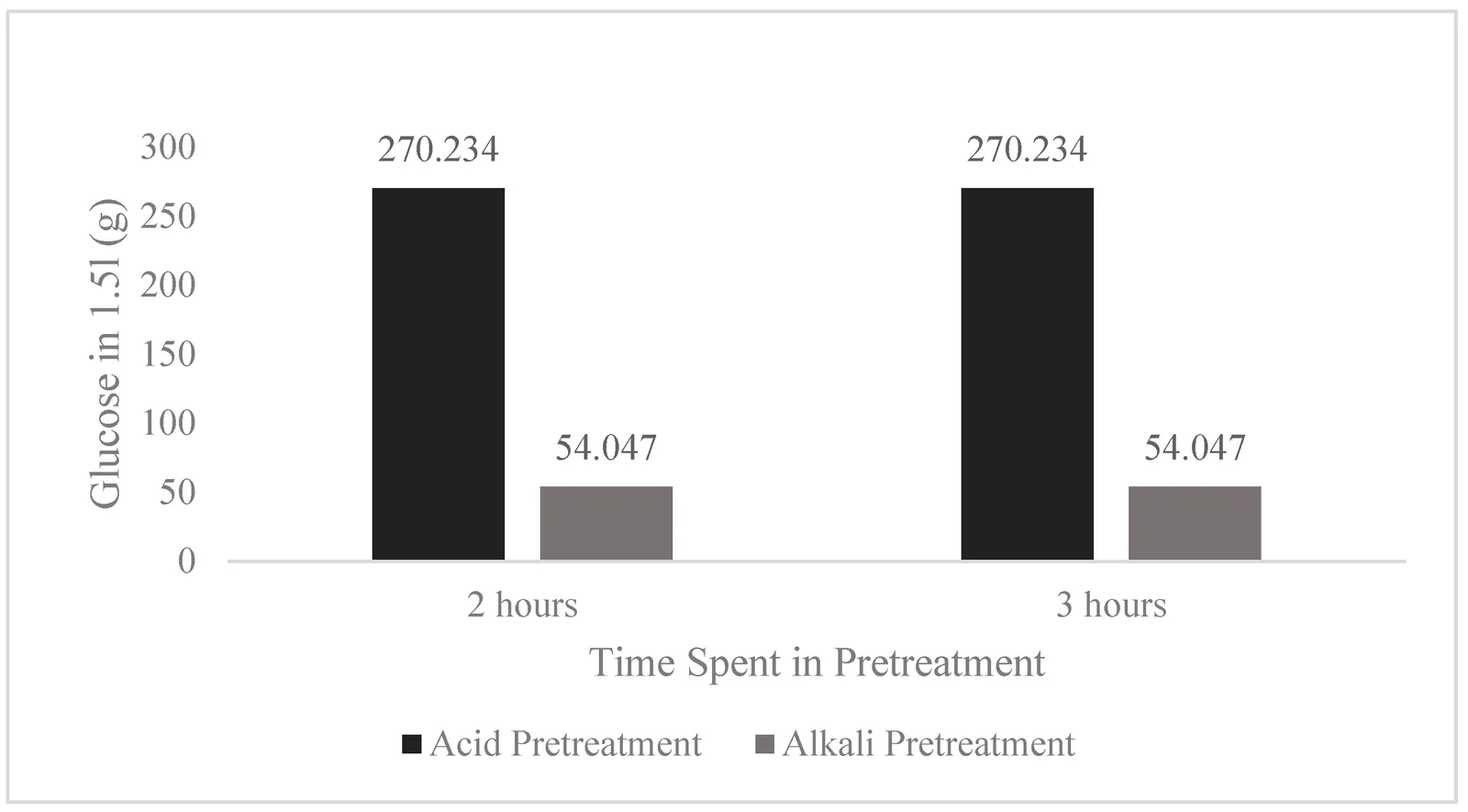
Calculated effect of time on alkali and acid pretreatments and the amount of glucose produced.

**Figure 6:**
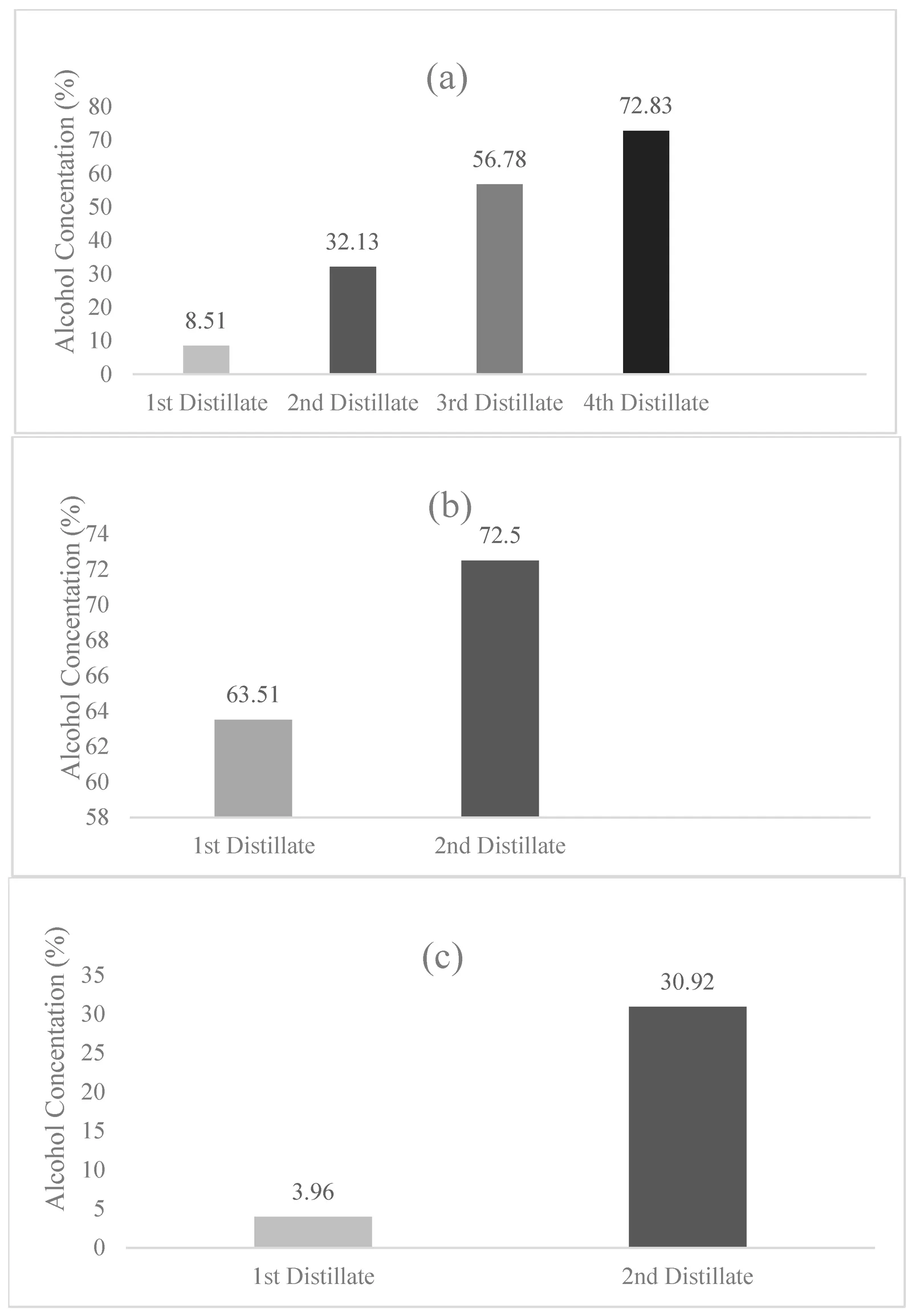
The amount of bioethanol formed, measured using the refractometer. (**a**) 1st distillate; (**b**) 2nd distillate; (**c**) 3rd distillate.

**Figure 7:**
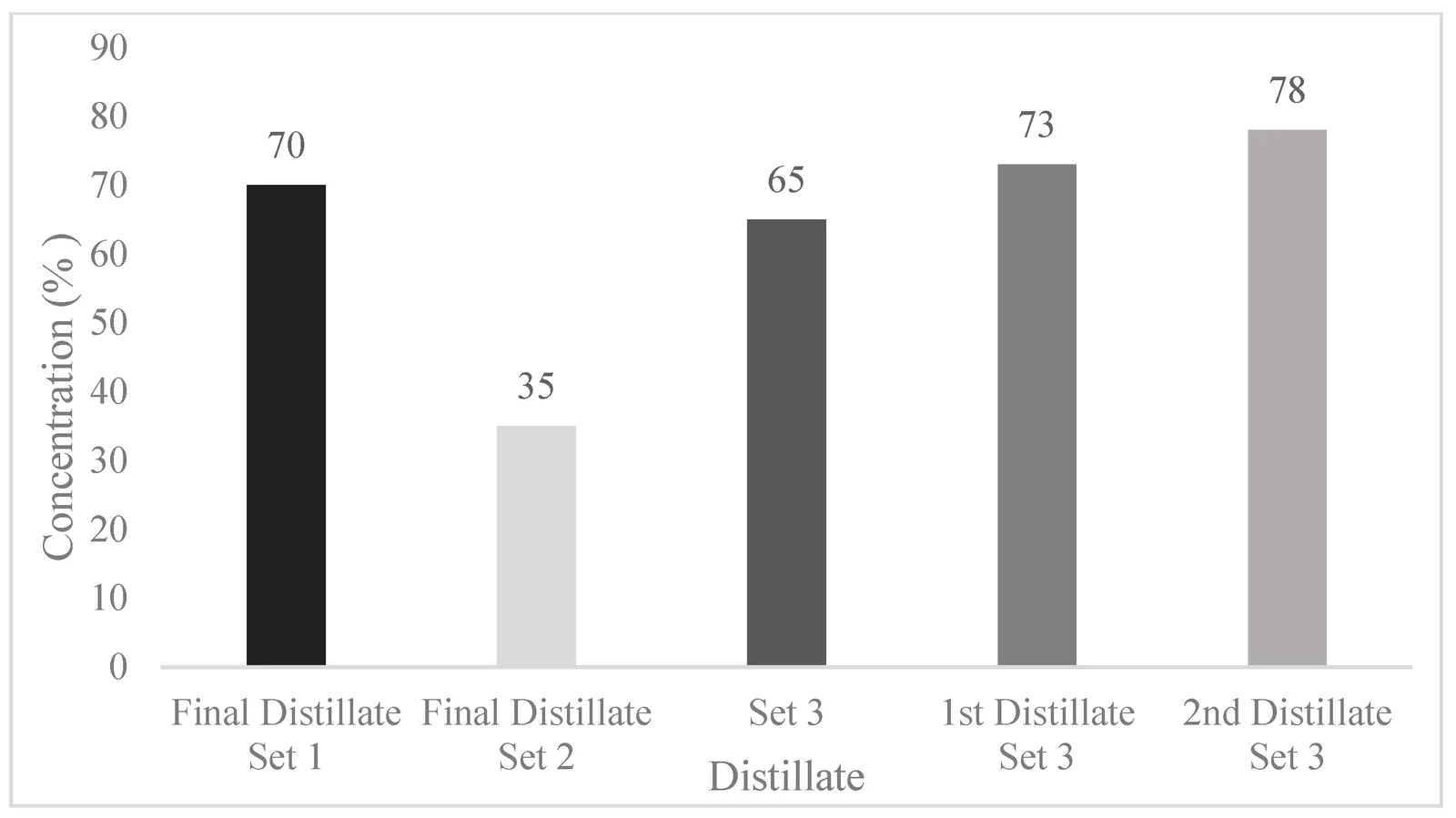
Alcohol concentration recorded with a hydrometer.

**Table 1:** Materials and chemicals used in this project.

Materials and Chemicals
Chempedak Peel
Sulfuric Acid
Sodium Hydroxide
Sodium Citrate Dihydrate
Citric Acid
Cellulase
Brewing Yeast (*Saccharomyces cerevisiae)*

**Table 2:** Chempedak peel sugar composition [13].

Chempedak Composition	Weight (%)
Total sugars	19.750 ± 0.160
Cellulose	27.750 ± 0.060
Starch	4.120 ± 0.020

**Table 3:** Maximum ethanol yield.

Composition	Calculated Values
Glucose (in 300 g of Chempedak)	154.860 g
Weight of Ethanol	79.197 g
Volume of Ethanol	100.377 mL

**Table 4:** Yield efficiency.

Composition	Volume (mL)
Maximum Ethanol Volume	100.377
Actual Ethanol Volume	29.930
Yield Efficiency	29.818%

Maximum Ethanol Volume—maximum theoretical amount of ethanol to be extracted. Actual Ethanol Volume—total amount of ethanol extracted.

## Data Availability

The original contributions presented in this study are included in the article. Further inquiries can be directed to the corresponding author.
